# Protocol for the development and validation of a risk prediction model for stillbirths from 35 weeks gestation in Australia

**DOI:** 10.1186/s41512-020-00089-w

**Published:** 2020-12-16

**Authors:** Jessica K. Sexton, Michael Coory, Sailesh Kumar, Gordon Smith, Adrienne Gordon, Georgina Chambers, Gavin Pereira, Camille Raynes-Greenow, Lisa Hilder, Philippa Middleton, Anneka Bowman, Scott N. Lieske, Kara Warrilow, Jonathan Morris, David Ellwood, Vicki Flenady

**Affiliations:** 1 NHMRC Centre of Research Excellence in Stillbirth, Mater Research Institute – University of Queensland, Level 3 Aubigny Place, Brisbane, 4101 Australia; 2 University of Melbourne, Melbourne, Australia; 3 School of Medicine, University of Queensland, Brisbane, Australia; 4 Department of Obstetrics & Gynaecology, University of Cambridge, Cambridge, UK; 5 Sydney Medical School, University of Sydney, Sydney, Australia; 6 Royal Prince Alfred Hospital, Sydney, Australia; 7 University of New South Wales, Sydney, Australia; 8 School of Public Health, Curtin University, Perth, Australia; 9 Centre for Fertility and Health, Norwegian Institute of Public Health, Oslo, Norway; 10 Telelethon Kids Institute, Perth Children’s Hospital, Perth, Australia; 11 School of Public Health, The University of Sydney, Sydney, Australia; 12 National Perinatal Epidemiology and Statistics Unit, Centre for Big Data Research in Health and School of Women’s and Children’s Health, University of New South Wales, Sydney, Australia; 13 South Australian Health and Medical Research Institute, SAHMRI Women and Kids, Adelaide, Australia; 14 School of Medicine, The University of Adelaide, Adelaide, Australia; 15 University of Queensland, Brisbane, Australia; 16 Women and Babies Research, The University of Sydney Northern Clinical School, St. Leonards, Australia; 17 Northern Sydney Local Health District, Kolling Institute, Sydney, Australia; 18 Department of Obstetrics and Gynaecology, Royal North Shore Hospital, Northern Sydney Local Health District, Sydney, Australia; 19 School of Medicine, Griffith University, Southport, Australia

**Keywords:** Fetal death, Stillbirth, Prediction, Prognostic, Risk, Obstetrics, Perinatal, AUROC

## Abstract

**Background:**

Despite advances in the care of women and their babies in the past century, an estimated 1.7 million babies are born still each year throughout the world. A robust method to estimate a pregnant woman’s individualized risk of late-pregnancy stillbirth is needed to inform decision-making around the timing of birth to reduce the risk of stillbirth from 35 weeks of gestation in Australia, a high-resource setting.

**Methods:**

This is a protocol for a cross-sectional study of all late-pregnancy births in Australia (2005–2015) from 35 weeks of gestation including 5188 stillbirths among 3.1 million births at an estimated rate of 1.7 stillbirths per 1000 births. A multivariable logistic regression model will be developed in line with current ***T****ransparent* ***R****eporting of a multivariable prediction model for* ***I****ndividual* ***P****rognosis or* ***D****iagnosis* (TRIPOD) guidelines to estimate the gestation-specific probability of stillbirth with prediction intervals. Candidate predictors were identified from systematic reviews and clinical consultation and will be described through univariable regression analysis. To generate a final model, elimination by backward stepwise multivariable logistic regression will be performed. The model will be internally validated using bootstrapping with 1000 repetitions and externally validated using a temporally unique dataset. Overall model performance will be assessed with *R*^*2*^, calibration, and discrimination. Calibration will be reported using a calibration plot with 95% confidence intervals (*α* = 0.05). Discrimination will be measured by the *C-*statistic and area underneath the receiver-operator curves. Clinical usefulness will be reported as positive and negative predictive values, and a decision curve analysis will be considered.

**Discussion:**

A robust method to predict a pregnant woman’s individualized risk of late-pregnancy stillbirth is needed to inform timely, appropriate care to reduce stillbirth. Among existing prediction models designed for obstetric use, few have been subject to internal and external validation and many fail to meet recommended reporting standards. In developing a risk prediction model for late-gestation stillbirth with both providers and pregnant women in mind, we endeavor to develop a validated model for clinical use in Australia that meets current reporting standards.

**Supplementary Information:**

The online version contains supplementary material available at 10.1186/s41512-020-00089-w.

## Background

Prevention of stillbirth remains one of the greatest challenges in modern maternity care. Globally, one in every 137 pregnancies that reach 20 weeks’ gestation will result in a stillborn child [1, 2]. Despite advances in the care of women and their babies in the past century, an estimated 1.7 million babies die before birth each year throughout the world [3]. The 2016 Lancet Ending Preventable Stillbirths series highlighted differences in rates of late stillbirth (≥ 28 weeks) between high-income countries ranging from 1.7 per 1000 to 8.8 per 1000 births [4]. Australia is a high-income country where over 2000 families each year—six families each day—have a stillbirth, and there has been no improvement in stillbirth rates among late pregnancy stillbirths for over 20 years [5, 6]. Among women who were born elsewhere [7, 8], women with lower socioeconomic status [9], and women who identify as Aboriginal and Torres Strait Islander [10], the risk of stillbirth is higher [4, 11]. Failure to identify and appropriately care for women with risk factors for stillbirth contributes to 20–50% of preventable stillbirths, which has the potential to avoid 400 stillbirths each year for Australian families [12–14].

Detecting women at risk for stillbirth is challenging. In the absence of a tool to assess a pregnant woman’s individualized risk of late-pregnancy stillbirth, we rely on generalized, population-level information. Awareness of risk factors that increase the risk of stillbirth at or near term is a necessary first step in improving care and to ultimately reduce the number of stillbirths. Despite a high proportion of unexplained stillbirths between 39 and 41 weeks of gestation, many women who have a stillbirth have one or more risk factors that are often unrecognized [15].

Around 38 weeks of gestation, the risk of stillbirth increases overall and varies by maternal and clinical characteristics while the decision on whether to intervene becomes more challenging [5, 10, 16, 17]. The balance between benefit and harm is complicated by potentially avoiding a stillbirth at the risk of neonatal morbidity [18]. A robust prediction model to assess a woman’s individualized risk of late-pregnancy stillbirth has the potential to alleviate some interventional uncertainty by informing antenatal care and decision-making around the timing of birth.

A key limitation of developing a late-gestation stillbirth risk prediction model for clinical use is the lack of high-quality data from a complete population. With recent quality improvements for population-level data in Australia, it is now possible to leverage population-based data to develop, internally validate, and externally validate a model to predict potentially preventable and rare pregnancy outcomes [19]. Therefore, the objective of this study is to develop and validate a prognostic model for late-pregnancy stillbirth risk that is designed to inform decision-making around the timing of birth.

## Methods

### Aim

We endeavor to develop multivariable logistic regression prediction models to estimate the risk of late-pregnancy stillbirth from 35 weeks of gestation using a national dataset of all births in Australia (2005–2015) to ultimately inform decision-making around the timing of birth for women who reside in Australia.

### Study design

This is a protocol for a cross-sectional study using the total population of singleton term gestation births in Australia (2005–2015) derived from the National Perinatal Data Collection (NPDC) (1998–2015) [11, 20]. The dataset includes 5188 stillbirths among 3.1 million births at an estimated rate of 1.7 stillbirths per 1000 births [11]. Multiple pregnancies, congenital abnormalities, and babies missing gestational age information will be excluded. A congenital abnormality is defined as a stillbirth classified as code 0100 “Congenital Abnormality” using the Perinatal Society of Australia and New Zealand (PSANZ) Perinatal Death Classification System [21]. A completed Compliance with Transparent Reporting of a multivariable prediction model for Individual Prognosis or Diagnosis (TRIPOD) checklist is available in supplementary materials (Supplementary Table 1).

### Sample size

To ensure the development of a robust prediction model for each week gestation from 35 weeks, sample size calculations recommended by Riley et al. are provided for stillbirth as a binary outcome to (B1) estimate overall outcome proportion with precision, (B2) target a small mean absolute prediction error, (B3) target a shrinkage factor of 0.9, and (B4) target small optimism of 0.05 in the apparent *R*^2^ [22]. Based on these criteria, the population derived from the NPDC is expected to be sufficient and is detailed below.

Stata 16.0 procedure pmsampsize was used for criteria B1, B3, and B4 where anticipated *R*^2^ value is 0.003 with a maximum of 25 parameters (candidate risk factors), and the overall proportion of stillbirth is 0.0017 and derived from the estimated stillbirth rate of 1.7 stillbirths per 1000 births in our study population [22, 23]:

$$ \mathrm{pmsampsize},\mathrm{type}\left(\mathrm{b}\right)\ \mathrm{rsquared}(0.003)\ \mathrm{parameters}(25)\mathrm{prevalence}(0.0017) $$

This indicates that at least 74,875 births are required, corresponding to 128 events (where the prevalence of stillbirth is 0.0017) and events per candidate predictor parameter of 5.09.

For criteria B2, we applied the mean absolute prediction error (MAPE) formula at a value of 0.050 for the anticipated outcome proportion (0.0017) and 25 candidate predictor parameters. This indicated a required total of 92 participants in the development dataset at a MAPE of 0.05 or 494 participants at a MAPE of 0.02.

### Data source

All births with gestational age information from 35 weeks of gestation in Australia (2005–2015) will be included. Data will be made available via the AIHW Maternal and Perinatal Health Unit. Further information on available data items and reporting can be found in the supplementary materials (Supplementary Table 2). The NPDC is a national population-based cross-sectional collection of data for all pregnancies and births established in 1991 [24]. All births from the 6 states and 2 territories of Australia are reported as part of the NPDC and include Queensland (QLD), New South Wales (NSW), Australian Capital Territory (ACT), Victoria (VIC), South Australia (SA), Tasmania (TAS), Western Australia (WA), and Northern Territory (NT) (Table 1). Perinatal data are collected for each birth in each state and territory, usually by midwives and other birth attendants [11]. The data is collated by the relevant state or territory health department and a standard de-identified extract is provided to the AIHW on an annual basis to form the NPDC [11]. Stillbirths in Australia are defined by the PSANZ as fetal deaths from gestational age of at least 20 weeks or birthweight of at least 400 g, except in Victoria and Western Australia, where births are included if gestational age is at least 20 weeks or, if gestation is unknown, birthweight is at least 400 g [11, 21].

**Table 1:** All births in Australia from 35 weeks of gestation, 2005–2015

Jurisdiction	Total (*n*)	Stillbirths (*n*)	Livebirth (*n*)	Stillbirth rate (per 1000)
NSW	1,021,491	1758	1,019,289	1.7
VIC	716,145	1161	714,486	1.6
QLD	645,416	1087	643,888	1.7
WA	336,532	517	335,808	1.5
SA	209,873	320	209,351	1.5
TAS	64,418	112	64,279	1.7
ACT	61,751	138	61,580	2.2
NT	40,723	95	40,592	2.3
Overall	3096349	5188	3089273	1.7

### Model development

**Fig. 1:**
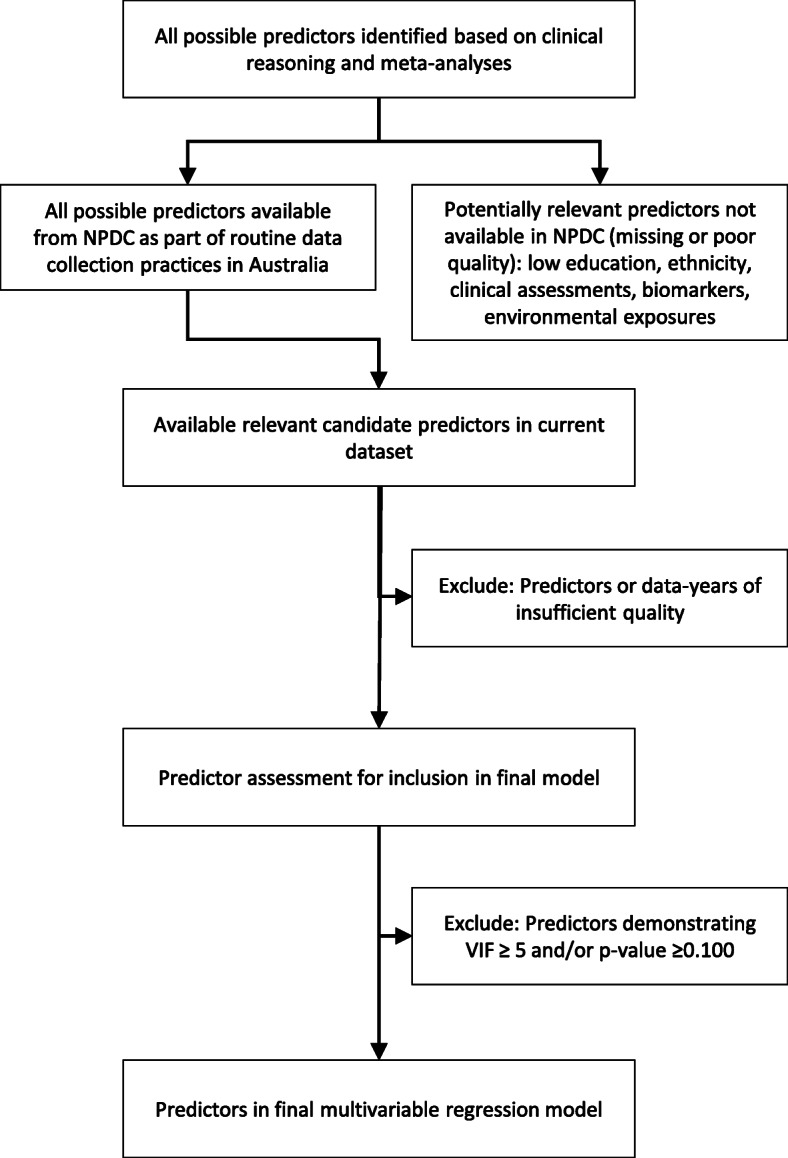
Selection of predictors in a study developing a multivariable logistic regression model for stillbirth

Established characteristics and conditions associated with an increased risk of stillbirth will be considered as candidate predictors [16, 25–27]. The predictor selection process is illustrated in Fig. 1. Reference group coding will be informed by literature and existing reporting recommendations. Frequencies (%) will be presented for categorical variables and for all missing data (further information on the handling of missing data described below). For normally distributed continuous variables, the mean and standard deviation will be reported. For continuous variables demonstrating skewed distributions, median and IQR will be reported. For all continuous variables, minimum and maximum will be presented. If clinically appropriate and statistically justifiable, independent continuous variables will be converted to groups according to published guidelines and recommendations [11, 28].

Univariable logistic regression models will be developed first for all gestations to explore individual prognostic factors where the outcome (stillbirth) is binary and the prognostic factors are either continuous or categorical. Univariable models will only be used to provide context to the final multivariable logistic regression model. Variance inflation factor (VIF) will be performed prior to fitting the final multivariable regression model to identify collinearity where VIF < 4 indicates a low correlation, VIF between 5 and 10 indicates a high correlation, and VIF above 10 indicates multicollinearity [29]. Candidate predictors demonstrating multi- or collinearity with VIF ≥ 5 will be reviewed through clinical consultation to ultimately select candidate predictor for inclusion in the final model. Backward stepwise elimination in a multivariable logistic regression model will be applied to remove non-significant factors with *p* values greater than 0.100 in line with Akaike’s information criterion [30]. Finally, the risk prediction model will be applied and fully validated for each week’s gestation from 35 weeks (six total models: 35, 36, 37, 38, 40, and 41+ weeks).

### Missing data

Missing data for predictors is most likely to result from failed reporting for all births in specific years by jurisdictions (see Supplementary Table 2 for comments on missing data). Data-years where reporting of candidature predictors may be excluded if missing data exceeds 5% for the total population [31, 32].

If clinically appropriate, a “hot deck” formula for multiple imputations will be considered for predictors with greater than 5% missing values where a substitute value will be imputed from another dataset [32–34]. For candidate predictors with fewer or equal to 5% missing values, missing values for categorical predictors will be treated as null or “no” and continuous predictors will be recorded as a mean value. No births will be excluded due to missing candidate predictor data except for those missing gestational age information.

### Validation

Final gestation-specific models will be subject to temporal internal and external validation. Population characteristics and performance measures will be reported for all individual models [35]. Internal validation will be performed using bootstrapping with 1000 repetitions [36]. Summary stillbirth rates will be reported for the bootstrapped sample. Final models will be externally validated using data derived from study years not used for model development [37].

### Model performance

The performance of development and validation datasets will be assessed via overall performance (*R*^2^), calibration, discrimination, and clinical performance will be assessed through positive predictive value (PPV) and negative predictive value (NPV). A fixed false-positive cutoff of 10% will be used for PPV and NPV [38].

Calibration characterizes model performance in terms of agreement between predicted (expected) risk and observed risk and is reported using a calibration plot [39]. An intercept of zero and ratio of observed and expected equal to one (O/E = 1) is defined as the best possible calibration [40]. Calibration plots will contain 95% confidence intervals to infer the degree of calibration between observed outcomes and predictions.

Discrimination is defined as the model’s ability to distinguish stillbirths and non-stillbirths and will be measured via calculation of the *C* statistic and receiver operator characteristic (ROC) curve. A ROC curve is used to assess the performance of a categorical classifier and is a plot of sensitivity (true positive rate) versus 1-specificity (false positive rate) where different points on the curve correspond to different cutoff points used to designate positive identification/classification [41].. Using the ROC curve, the performance of the predictors will be further quantified by calculating the area under the curve or AUC. The AUC score range is 0.0–1.0, where a score of 0.5 can be equated to a “coin flip”, 0.0 is perfectly inaccurate, and 1.0 is perfectly accurate [42]. A non-parametric comparison of AUC will be performed using the Mann-Whitney *U* statistic for individual gestational age models [26].

In addition to calibration and discrimination, PPV and NPV will be reported to characterize clinical usefulness. A decision curve analysis will be considered to characterize potential decision thresholds [43].

## Discussion

Prediction models designed for obstetrics hold enormous promise. However, unlike other clinical prediction models, we do not yet understand whether their application improves birth outcomes [44]. With many models for adverse pregnancy outcomes being developed through various approaches, it is inevitable that only a minority have been subject to full internal and external validation and many fail to meet recommended reporting standards. By utilizing a population-based, individual-level dataset, our study is expected to provide a sufficient sample size of singleton stillbirths and births to develop and validate gestation-specific prediction models that can be translated into clinical tools or decision aids.

There have been attempts to develop risk prediction models for stillbirth, yet none are designed to predict stillbirth risk at- or near-term or use a population-level data source for singleton pregnancies in a high-income setting [45]. Among existing prediction models designed for obstetrics, logistic regression models are widely utilized [45]. Yerlikaya et al. reported a prediction model for stillbirth with low predictive accuracy beyond the early term period [46]. Trudell et al. reported a clinical prediction tool for antenatal testing with the modest discrimination for stillbirth at or beyond 32 weeks’ gestation that included risk factors such as maternal age, African-American/Black race, nulliparity, body mass index, smoking, chronic hypertension, and pre-gestational diabetes [36]. Although there is a growing interest in algorithmic methods such as machine learning, evidence suggests that performance is highly comparative to statistical modeling [47, 48]. Regarding approaches to validation, the most commonly used methods include split-sample, bootstrap, and cross-validation. Bootstrapping tends to demonstrate increased variability and split-sampling often results in unreliable assessments of model performance. A cross-validation is an effective approach for validating a prediction model for low-prevalence obstetric outcomes like stillbirth due to stability and ability to use a larger part of the study sample for model development [42, 49]. Cross-validation is an extension of split-sample validation that uses a larger part of the sample for model development (> 80% vs. 50%) [39]. While not the most computationally efficient approach, the bootstrap repeated procedure is ideal and expected to produce stable results while conserving the complete study population for validation [22, 36, 50]. In our proposed validation design, a temporal approach to externally validate the model will be explored. While this is not considered a “fully independent external validation,” it is expected to provide an additional layer of assessment not yet reported for any existing stillbirth prediction model.

While there are numerous benefits to utilizing large observational datasets for the development of prediction models—particularly for rare pregnancy outcomes and multiple pregnancies, there are certain limitations [51]. Completeness of routinely reported variables and potentially relevant risk factors not captured by the NPDC, such as maternal ethnicity, will have an impact on the final model. Missing data for risk factors used in a prediction model will be vulnerable to misclassification due to reporting evolution over time. While clinical definitions have largely remained consistent from 1998 to 2015, some data items for certain years have changed from voluntary to required. The impact of these changes over time on classification is not yet documented and will be assessed through a supplementary sensitivity analysis. Certain variables collected by NPDC that are not available for release due to quality issues include maternal asthma, type of assisted reproductive therapy, fetal growth restriction, and other pregnancy-specific medical conditions. Environmental exposures are not currently captured by the NPDC, and other spatial risk factors cannot be explored due to sensitivity restrictions. However, most key risk factors identified in literature and informed by background clinical knowledge will be considered and are expected to produce a full prediction model for stillbirth using routinely collected data without attempting to identify new predictors or using biomarkers. Future studies should consider exploring the care pathway and risk management of multiple pregnancies and unique risk factors (including maternal pregnancy conditions).

Lastly, subsequent pregnancy outcomes depend heavily on the outcome of previous pregnancies where each birth is not independent of births [52–54]. An anticipated complication of our analysis that will impact on the interpretation of results is the absence of a unique identifier for mothers to account for potential clustering. Parity will be assessed to distinguish first versus subsequent births [55], but the lack of independence of births in our models will be limited. There are recommendations for the generalized estimating equation approach, but will not be possible due to an inability to appropriately cluster pregnancies according to unique mothers [55, 56].

Using known predictors from routine population-level data, we endeavor to develop a validated risk prediction model for late-gestation stillbirth for clinical use in Australia with both providers and pregnant women in mind that meets all TRIPOD standards and recommendations [57]. Such a prediction model could be used in a narrow or broad impact analysis that explores decision rules to reduce stillbirth by improving decision-making around the timing of birth [43, 49].

## Supplementary Information

**Additional file 1.**

**Additional file 2.**

## Data Availability

Study outputs including full model details will be published in a peer-reviewed journal; however, the dataset is not publicly available due to sensitivity and individual privacy protection restrictions as stipulated by human research ethics. Study data can be accessed through a formal request from the AIHW (https://www.aihw.gov.au/our-services/data-on-request).

## Abbreviations

TRIPOD: Transparent Reporting of a multivariable logistic regression model for Individual Prognosis or Diagnosis
AIHW: Australian Institute of Health and Welfare
NPDC: National Perinatal Data Collection
ROC: Area under the receiver-operator curve
HREC: Human research ethics committee(s)
PSANZ: Perinatal Society of Australia and New Zealand
NHMRC: National Health and Medical Research Council
ASGS: Remoteness Area as per the Australian Statistical Geography Standard
ASGC: Australian Standard Geographical Classification (ASGC)
SEIFA: Socio-Economic Indexes for Areas
IRSD: Index of Relative Socio-Economic Disadvantage
VIF: Variance inflation factor
NT: Northern Territory
QLD: Queensland
NSW: New South Wales
VIC: Victoria
ACT: Australian Capital Territory
TAS: Tasmania
SA: South Australia
WA: Western Australia
