# Supplementary material for: Protocol for the development and validation of a risk prediction model for stillbirths from 35 weeks gestation in Australia
Source: Diagn Progn Res. 2020 Dec 16;4:21. doi: 10.1186/s41512-020-00089-w (PMC7739473; doi:10.1186/s41512-020-00089-w)
Supplement: Supplementary file 2 — Additional file 2. [file 41512_2020_89_MOESM2_ESM.docx]

**Table S2. Original dataset characteristics of candidate predictors for all births in Australia, 2005-2015.**

| Candidate predictor | Reported value | Reported label | Missing data comments |
| --- | --- | --- | --- |
| Hospital sector | 1 | Public | Data only collected from 2001. Data were not available for VIC in 2009. |
|  | 2 | Private |  |
|  | 8 | Not applicable (e.g., home birth) |  |
|  | Missing | Jurisdiction did not provide any data |  |
|  | 9 | Not stated |  |
| Maternal age in years | <19 | Less than 19 |  |
|  | 19–44 | Single values |  |
|  | >=45 | 45 and over |  |
|  | 99 | Not stated |  |
| Maternal Indigenous status | 1 | Indigenous - Aboriginal and/or Torres Strait Islander | Data only collected from 1999. Data were not available for TAS until 2005. |
|  | 2 | Non-Indigenous |  |
|  | Missing | Jurisdiction did not provide any data |  |
|  | 9 | Not stated |  |
| Maternal country of birth | ASCSS, SACC codes | ASCSS, SACC 1st edition, SACC 2nd edition, SACC 2011 or pre-arranged groupings | Between 1998 and 2003, QLD were unable to provide data for the NPDC according to the ASCCSS or the SACC, and instead provided grouped data.  Between 1998 and 1999 all jurisdictions (with the exception of QLD) provided data according to the ASCCSS classification.  In 2000, NT provided data using the SACC first editions, while other jurisdiction provided data using the ASCCSS or arranged groupings (QLD).  In 2001, VIC, WA, TAS and ACT provided data using the ASCCSS, while NSW, SA, and NT provided using the SACC first edition.  Between 2002 and 2003, VIC and TAS provided data using the ASCCSS, QLD provided the pre-arranged groupings and all other jurisdictions provided data using the SACC first edition.  In 2004, VIC and TAS provided data using the ASCCSS, and all other jurisdictions provided data using the SACC first edition.  Between 2005 and 2008, VIC provided data using the ASCCSS and all other jurisdictions provided data using the SACC first edition.  Between 2009 and 2011, all jurisdictions provided data according to the SACC second edition.  From 2012, all jurisdictions provided data using SACC 2011. |
| Pre-existing diabetes during pregnancy | 0 | None/not stated | Between 1998 and 2013, this was a voluntary, non-standard data item. Data quality and definitions used varies significantly by state/territory and year. The AIHW advises caution when using this data item. From 2014, data are collected as part of the NPDC NBEDS. Please note that 2014-15 data are not comparable with data from previous years.  Data are not available for PREDIAB in the states and territories for the years listed below:  – ACT in 2000 and 2002  – TASas between 2000 and 2004  – VIC between 2009 and 2015  – WA in 2013  – NT in 1998 and 2000 |
|  | 1 | Pre-existing diabetes |  |
|  | Missing | Jurisdiction did not provide any data |  |
| Gestational diabetes | 0 | None/not stated | Between 1998 and 2013, this was a voluntary, non-standard data item. Data quality and definitions used varies significantly by state/territory and year. The AIHW advises caution when using this data item. From 2014, data are collected as part of the NPDC NBEDS. Please note that 2014-15 data are not comparable with data from previous years.  Data are not available for GESTDIAB in the states and territories for the years listed below:  – ACT in 2000 and 2002  – VIC between 2009 and 2015  – WA in 2013  – NT in 1998 and 2000  – TAS between 2000 and 2004 |
|  | 1 | Pre-existing diabetes |  |
|  | 2 | Gestational diabetes mellitus (GDM) |  |
|  | Missing | Jurisdiction did not provide any data |  |
| Chronic hypertension during pregnancy | 0 | None/not stated | Between 1998 and 2013, this was a voluntary, non-standard data item. Data quality and definitions used varies significantly by state/territory and year. The AIHW advises caution when using this data item. From 2014, data are collected as part of the NPDC NBEDS. Please note that 2014-15 data are not comparable with data from previous years.  Data are not available for PREHYPT in the states and territories for the years listed below:  – ACT in 2000 and 2002  – TAS between 2000 and 2004  – VIC between 2009 and 2015  – WA in 2013  – NT in 1998 and 2000 |
|  | 1 | Chronic hypertension |  |
|  | Missing | Jurisdiction did not provide any data |  |
| Maternal medical conditions: Essential hypertension | 0 | None/not stated | Between 1998 and 2013, this was a voluntary, non-standard data item. Data quality and definitions used varies significantly by state/territory and year. The AIHW advises caution when using this data item. From 2014, data are collected as part of the NPDC NBEDS. Please note that 2014-15 data are not comparable with data from previous years.  Data are not available for GESTHYPT in the states and territories for the years listed below:  – ACT between 2000 and 2002  – VIC between 2009 and 2015  – WA in 2013  – NT in 2000, 2009 and 2010  – TAS between 2000 and 2004 |
|  | 1 | Gestational hypertension |  |
|  | Missing | Jurisdiction did not provide any data |  |
| 2006 Socio-Economic Indexes for Areas (SEIFA) Index of Relative Socio-Economic Disadvantage (IRSD) | 1 | Quintile 1 (most disadvantaged) | This data item is still being investigated. AIHW may be able to provide data between 2007 and 2011. Data quality have been assessed between 1998 and 2006, and were not sufficient for release, therefore data are not available in these years. |
|  | 2 | Quintile 2 |  |
|  | 3 | Quintile 3 |  |
|  | 4 | Quintile 4 |  |
|  | 5 | Quintile 5 (least disadvantaged) |  |
|  | 9 | Not stated |  |
| 2011 Socio-Economic Indexes for Areas (SEIFA) Index of Relative Socio-Economic Disadvantage (IRSD) | 1 | Quintile 1 (most disadvantaged) | Data are available from 2012. |
|  | 2 | Quintile 2 |  |
|  | 3 | Quintile 3 |  |
|  | 4 | Quintile 4 |  |
|  | 5 | Quintile 5 (least disadvantaged) |  |
|  | Missing | Jurisdiction did not provide any data |  |
|  | 9 | Not stated |  |
| Remoteness Area as per the Australian Standard Geographical Classification (ASGC) | 0 | Major cities | Data quality have been assessed between 1998 and 2006, and were not sufficient for release, therefore data are only available from 2007. |
|  | 1 | Inner regional |  |
|  | 2 | Outer regional |  |
|  | 3 | Remote |  |
|  | 4 | Very remote |  |
|  | Missing | Jurisdiction did not provide any data |  |
|  | 9 | Not stated |  |
| Remoteness Area as per the Australian Statistical Geography Standard (ASGS) | 0 | Major cities | Data are available from 2012. |
|  | 1 | Inner regional |  |
|  | 2 | Outer regional |  |
|  | 3 | Remote |  |
|  | 4 | Very remote |  |
|  | Missing | Jurisdiction did not provide any data |  |
|  | Blank | Not able to be assigned, non-Australian resident and not stated |  |
| Marital status | 1 | Never married | This is a voluntary, non-standard data item. Data quality varies significantly by state/territory and year. The AIHW advises caution given the high level of not stated values for some jurisdictions in some years. Data are not available for MAR_STAT in the states and territories for the years listed below:  – TAS between 2005 and 2009  – WA between 2013 and 2015  – VIC in 2009 |
|  | 2 | Widowed, divorced, separated |  |
|  | 3 | Married (including de facto) |  |
|  | Missing | Jurisdiction did not provide any data |  |
|  | 9 | Not stated |  |
| Total number of previous pregnancies resulting in a livebirth or a stillbirth | 0-4 | Single values | In 2009, VIC did not provide any parity data. |
|  | >=5 | 5 and over (grouped) |  |
|  | Missing | Jurisdiction did not provide any data |  |
|  | 9 | Not stated |  |
| Previous caesarean sections | 0-4 | Single values | Data quality have been assessed in 1999, and were not sufficient for release, therefore data are only available from 2000. |
|  | >=5 | 5 and over (grouped) |  |
|  | Missing | Jurisdiction did not provide any data |  |
|  | 9 | Not stated |  |
| Caesarean section for last birth | 1 | Yes | Data only collected from 2004. Data are not available in the states and territories for the years listed below:  – ACT in 2004 and 2005  – TAS in 2004  – VIC in between 2007 and 2009  – WA in 2013 |
|  | 2 | No |  |
|  | 7 | Not applicable |  |
|  | Missing | Jurisdiction did not provide any data |  |
|  | 9 | Not stated |  |
| Previous pregnancies resulting in stillbirths | 0-1 | Single values | This is a voluntary, non-standard data item. Data quality and definitions used varies significantly by state/territory and year. The AIHW advises caution given the high level of not stated values for some jurisdictions in some years. Data are not available in the states and territories for the years listed below:  – NSW in any year  – SA between 2000 and 2015  – Vic in 2009  – WA between 2013 and 2015 |
|  | >=2 | 2 and over (grouped) |  |
|  | Missing | Jurisdiction did not provide any data |  |
|  | 9 | Not stated |  |
| Smoking status during pregnancy | 1 | Smoked | This is a voluntary, non-standard data item. Data quality and definitions used varies significantly by state/territory and year. The AIHW advises caution when using this data item. Data are not available until 2001, or in the states and territories for the years listed below:  – VIC between 1998 and 2009  – QLD before 1 July 2005  – TAS between 1998 and 2004 |
|  | 2 | Did not smoke |  |
|  | Missing | Jurisdiction did not provide any data |  |
|  | 9 | Not stated |  |
| Smoking status during first twenty weeks of pregnancy | 1 | Smoked | Data are not available until 2010, or in the states and territories for the years listed below:  – NSW in 2010  – NT before 1 July 2010  – TAS between 2010 and 2012 |
|  | 2 | Did not smoke |  |
|  | Missing | Jurisdiction did not provide any data |  |
|  | 9 | Not stated |  |
| Smoking status after twenty weeks of pregnancy | 1 | Smoked | Data are not available until 2010, or in the states and territories for the years listed below:  – NSW in 2010  – NT before 1 July 2010  – TAS between 2010 and 2012 |
|  | 2 | Did not smoke |  |
|  | Missing | Jurisdiction did not provide any data |  |
|  | 9 | Not stated |  |
| Pre-pregnancy Body Mass Index (BMI) | ≥ 9.0 | Continuous values or rounded to the nearest 5 kg/m2 | This is a voluntary, non-standard data item. Data quality and definitions used varies significantly by state/territory and year. The AIHW advises caution when using this data item. Data are not available for BMI until 2008, or in the states and territories for the years listed below:  – NSW in any year  – ACT in 2008  – NT in 2008, 2009, 2011 or 2013  – VIC and TAS in 2008 and 2009  – WA between 2008 and 2010  NOTE: QLD (2008-2011) and ACT (2009-2011) provided data rounded to the nearest 5 kg/m2. |
|  | Missing | Jurisdiction did not provide any data |  |
|  | 99.9 | Not stated |  |
| Plurality | 1-8 | Single values |  |
|  | 9 | Not stated |  |
| Assisted reproduction technology flag | 0 | No | This is a voluntary, non-standard data item. Data quality and definitions used varies significantly by state/territory and year. The AIHW advises caution when using this data item. Data are not available between 1998 and 2007, or in the states and territories for the years listed below:  – NSW, SA and NT in any year  – VIC in 2009  – WA in 2007 and between 2013 and 2015 |
|  | 1 | Yes |  |
|  | missing | Jurisdiction did not provide any data |  |
|  | 9 | Not stated |  |
| Baby's birth order | 1-8 | Single values |  |
|  | 9 | Not stated |  |
| Baby's sex | 1 | Male |  |
|  | 2 | Female |  |
|  | 9 | Indeterminate and not stated |  |
